# Characterization of stress coping style in Senegalese sole (*Solea senegalensis*) juveniles and breeders for aquaculture

**DOI:** 10.1098/rsos.160495

**Published:** 2016-11-09

**Authors:** Z. Ibarra-Zatarain, E. Fatsini, S. Rey, O. Chereguini, I. Martin, I. Rasines, C. Alcaraz, N. Duncan

**Affiliations:** 1 IRTA, Sant Carles de la Ràpita, Carretera de Poble Nou, km 5.5, 43540 Sant Carles de la Ràpita, Tarragona, Spain; 2 CENIT, Centro Nayarita de Innovación y Transferencia de Tecnología, Av. Emilio M. González s/n., CP 63173. Tepic, México; 3 Institute of Aquaculture, University of Stirling, Stirling, Scotland FK9 4LA, UK; 4 Spanish Institute of Oceanography, Santander Oceanographic Centre, Promontorio San Martín, s/n. PO 240, 39004 Santander, Spain

**Keywords:** *Solea senegalensis*, stress coping styles, animal personality, behavioural syndromes, exploratory behaviour, operational behavioural screening test

## Abstract

The aim of this work was to characterize stress coping styles of Senegalese sole (*Solea senegalensis*) juveniles and breeders and to select an operational behavioural screening test (OBST) that can be used by the aquaculture industry to classify and select between behavioural phenotypes in order to improve production indicators. A total of 61 juveniles and 59 breeders were subjected to five individual behavioural tests and two grouping tests. At the end of the individual tests, all animals were blood sampled in order to measure cortisol, glucose and lactate. Three tests (restraining, new environment and confinement) characterized the stress coping style behaviour of Senegalese sole juveniles and breeders and demonstrated inter-individual consistency. Further, the tests when incorporated into a principal components analysis (PCA) (i) identified two principal axes of personality traits: ‘fearfulness-reactivity’ and ‘activity-exploration’, (ii) were representative of the physiological axis of stress coping style, and (iii) were validated by established group tests. This study proposed for the first time three individual coping style tests that reliably represented proactive and reactive personalities of Senegalese sole juveniles and breeders. In addition, the three proposed tests met some basic operational criteria (rapid testing, no special equipment and easy to apply and interpret) that could prove attractive for fish farmers to identify fish with a specific behaviour that gives advantages in the culture system and that could be used to establish selection-based breeding programmes to improve domestication and production.

## 1. Introduction

Individuals subjected to hazardous conditions exhibit a wide range of different individual responses or stress coping styles that range from proactive to reactive behaviours [1]. Compared with reactive individuals, proactive individuals are usually bolder and more active, take higher risk when faced with potential threats, but show lower flexibility and sensitivity to changes in the environment [1–5]. Physiologically, proactive fishes are characterized by a lower hypothalamus–pituitary–interrenal (HPI) axis activity compared with reactive fish, leading to lower post-stress levels of glucocorticoids (i.e. cortisol) [2,6]. The neuroendocrine system, which regulates the liberation of glucocorticoids hormones in fish, is functionally homologous to the hypothalamus–pituitary–adrenal axis (HPA) in mammals. Nevertheless, coping strategies may be influenced by life experience and contexts, such as confinement, predation, novel situations or others environmental parameters [4,6,7]. Therefore, reliable tests are essential in order to characterize the stress coping styles of different fish species [8].

The assessment of proactive and reactive individuals is of interest for aquaculture, in order to increase the productivity and to establish genetic breeding lines with improved growth, survival and resistance to diseases [9]. In this sense, several studies have shown the significance of both behaviours in different fish species in wild conditions or kept in captivity. For instance, some authors found that proactive fish tended to grow faster [10,11], possessed a higher immune response [12] and a higher reproductive success [13,14], but relied on routines and presented a short life expectancy in the presence of predators [15]. On the other hand, reactive fish seemed to pay more attention to external stimuli, possessed high flexibility to changing environments [16,17] and presented higher anti-predatory responses [18]. Thus, proactive and reactive behaviours appear to play an important role in fish fitness and performance in captivity and could, therefore, influence productivity in aquaculture.

Different tests have been used to characterize fish stress coping style, often with the objective to assess stress coping style from an ecological perspective. These include evaluating the fish reaction when confronted by predators [19], observing the feeding behaviour [20] and assessing the latency to feed after disturbance [21], observing success and failure in fights [7], evaluating the competition for a food resource [12] and determining the willingness to take decisions [22]. Most of the previous examples were designed for an ecological approach and have been generally applied in experimental conditions that required specifically designed tanks with divisions and monitoring equipment, and all have taken long time periods to obtain results and demanded special technical skills to interpret the animal behaviour (‘*body language*’). These reasons make the cited tests difficult to apply to large numbers of fish in aquaculture and, for example, reactions in relation to predators are of questionable relevance to aquaculture. Therefore, operational behavioural screening tests (OBST) that can be quickly performed, with relatively few modifications of the rearing environment, easily achievable and with results that could be easily transformed into quantitative variables by farmers with minimal expertise, would be suitable for the aquaculture industry.

Senegalese sole (*Solea senegalensis*) is currently reared under intensive production systems in Spain, France and Portugal [23]. However, there still exist some bottlenecks that affect production such as mortalities during weaning, variable growth and poor juvenile quality and complete reproductive failure of first generation males (G1) reared in captivity to court females and fertilize eggs [23]. Therefore, the development of specific and reliable tests to characterize stress coping styles in juveniles and breeders of Senegalese sole may be useful for the aquaculture industry. Such OBST could be used to select individuals with particular behavioural characteristics that may give advantages for reproductive fitness or be used to establish a selection-based breeding programme in order to improve domestication and produce fingerlings with a specific behavioural trait. In this context, the aim of this study was (i) to confirm the existence of stress coping styles in juveniles and breeders, and (ii) to select some reliable tests to easily characterize the stress coping style behaviours of Senegalese sole juveniles and breeders reared in captivity.

## 2. Material and methods

### 2.1. Fish maintenance and identification

Senegalese sole juveniles (*n* = 61; weight = 46 ± 2 g; length = 15.2 ± 0.2 cm) and breeders (*n* = 59; weight = 1189 ± 50 g; length = 45.8 ± 0.7 cm) were used for this study. Juveniles were obtained from a commercial aquaculture farm (Stolt Seafarm, Galicia, Spain), and were acclimatized into IRTA facilities for three months prior to tests, while breeders were held in IRTA facilities for at least 5 years. During these periods prior to experiments, all fish were held under natural photoperiod and temperature conditions. Juveniles were housed in eight 500 l rectangular tanks, while breeders were stocked in four 13 m^3^ rectangular tanks. All tanks were located in a greenhouse structure and were connected to a recirculation system (IRTAmar®) to maintain a simulated natural water temperature (9–19°C: winter to summer) and oxygen (5–6 mg l^-1^) levels. Photoperiod was natural ranging from approximately light dark (L : D) 14 : 10 in the summer to 10 : 14 in the winter for breeders and juveniles. All individuals were tagged with a passive integrated transducer (PIT-ID-100 Unique, Trovan-Zeus, Madrid, Spain) for identification. Juveniles and breeders were fed ad libitum every morning (10.00) in the following manner: (i) juveniles: daily with balanced feed (LE-3 mm ELITE, Skretting Co.); and (ii) breeders: on Monday and Sunday balanced feed (Repro-Vitalis, LE-7 mm ELITE, Skretting Co.), on Wednesday cooked mussels (Sariego Intermares, Spain), and on Friday, marine polychaetes (Topsy-Baits, Holland). One hour after feeding, uneaten food was removed from all tanks to maintain optimal physico-chemical water conditions.

### 2.2. Stress coping styles tests

In order to not affect the reproductive cycle of Senegalese sole breeders, the individual and group coping style tests were first performed in the juveniles and subsequently in breeders. Further, the individual tests were performed simultaneously one after another to mimic the normal handling farming procedures [24]. For juveniles, individual coping style tests were performed from 23 to 26 July, and group tests from 13 to 17 August. For breeders, individual stress coping style tests were made from 6 to 10 October, and group tests from 6 to 11 November. The individual coping style tests, which were applied on randomly selected juveniles and breeders with the same behavioural criteria were: (i) restraining, (ii) evaluating the reaction to a new environment, (iii) evaluating the reaction to a confinement situation, (iv) flipping over the fish, and (v) inducing anaesthesia. Subsequently, a sample of blood was obtained from each anaesthetized individual to measure cortisol, glucose and lactate. The group tests consisted of: (i) novel object and (ii) risk taking.

### 2.3. Individual coping style tests

#### 2.3.1. Test 1: restraining test

Fish behaviour was evaluated by holding fish in a net in water (90 s) and then out of water in air (90 s). Two behavioural variables were evaluated in the restraining test: (i) the total activity time and (ii) the total number of escape attempts. (i) The total activity time was the time that fish spent moving inside the net, considered as the swimming activity inside the water, *NetActW*, or the contortions or shivers in the air outside the water, *NetActA*, and (ii) the total number of escape attempts was the number of body torsions resulting in an elevation of the body from the net, inside the water *NetEscW* or outside the water *NetEscA*. Selected variables were adapted from previous studies performed with this species [25–27], and other fish species, such as gilthead seabream *Sparus aurata* [28]. The abbreviations used for these tests indicate the parameter measured in the test, Net, net; Act, total activity time; W, water; A, Air (out of the water) and Esc, escape attempts.

#### 2.3.2. Test 2: new environment test

Once the first net test was completed, each fish was individually placed in a plastic tank that simulated a new environment. Dimensions of the tank were 56.5 × 36.5 × 30 cm for juveniles and 114 × 95 × 57 cm for breeders. Two behavioural parameters were registered during 5 min: (i) the latency time and (ii) the total activity time. (i) The latency time was the time from being introduced to the new environment until the first movement, *NewLat*, considered as the first moment when fish started to explore the new environment, if fish did not move at all during the 5 min period, then 300 s was recorded for statistical analysis [29], and (ii) the total activity time, *NewAct*, referring to the total time each fish spent swimming forward in the tank. During the test, observers stood completely stationary 1 m away from the tank to cause minimal disturbance to fish [30]. Tanks were provided with constant water that maintained more than 5–6 mg l^−1^ oxygen levels. Methodology was adapted from tests documented in bluegill sunfish *Lepomis macrochirus* [5], common sole *S. solea* [20] and stickleback *Gasterosteus aculeatus* [31]. The abbreviations used indicate the parameter measured, New, new environment; Lat, latency time and Act, total activity time.

#### 2.3.3. Test 3: confinement situation test

Fish were individually placed in a plastic tank that simulated a confinement situation. Dimensions of tanks were 25 × 14 × 8 cm for juveniles and 56 × 36 × 30 cm for breeders. Two behavioural parameters were registered for 5 min: (i) the latency time and (ii) total activity time. (i) The latency time was the time from being introduced into confinement until the first movement, *ConLat*, considered as the first moment that fish started to move, if fish did not move at all during the 5 min period, then 300 s was recorded for statistical analysis [29], and (ii) total activity time, *ConAct*, that was restricted to active locomotion against the walls of the confinement container. As for the previous test, observers stood stationary 1 m away from the container to not disturb fish. Methodological procedures were adapted from those documented in brown trout *Salmo trutta* [32] and rainbow trout *Oncorhynchus mikyss* [33]. The abbreviations used indicate the parameter measured, Con, confinement; Lat, latency time and Act, total activity time.

The number of opercula openings, *NumOper*, as a breathing rate approach, was counted and registered for juveniles during the first minute of this test [34].

#### 2.3.4. Test 4. flipping fish over

This test consisted in flipping fish over (eyes down position) and in recording the time (in seconds) the fish needed to recover its normal ventral position (*Flip*). Fish were laid on a rubber-foam carpet to cause minimal skin damage. Fish unable to recover normal ventral position after 3 min were turned over by hand and a maximum time of 180 s was assigned for statistical analysis. The selection of this test was following recommendations of the authors, who observed that during a sampling procedure, which involved flipping the fish, some fish recover or fought to recover the normal position faster than others.

#### 2.3.5. Test 5: anaesthesia induction

The time needed to induce three anaesthesia levels was determined in sole breeders. Sedation levels were selected in accordance with Schoettger & Julin [35] and were named as (i) light sedation *LSed*, characterized by the partial loss of reactivity; (ii) total sedation *TSed*, described as the impossibility to recover a normal position once turned over by hand; and (iii) deep anaesthesia *DSed*, when fish completely lost reflex to external stimuli. The anaesthetic agent consisted of tricaine methanesulfonate (MS-222; Acros-Organic, New Jersey, USA). Anaesthesia was prepared by adding 20 mg of MS-222 to 1 l of distilled water. A final dose of 60 µg l^−1^ in 45 l bath was used for the test [36]. This test was selected as authors observed that different soles reacted differently and apparently consistently in the anaesthesia bath (i.e. during repeated monthly sampling certain sole were always more difficult to anaesthetize than other sole) and on the basis that anaesthesia can induce differences in cortisol plasma levels, which is a good marker of stress coping styles [30,33], and was adapted from studies performed by Welker *et al*. [37] and Nordgreen *et al*. [38].

### 2.4. Group coping style tests

#### 2.4.1. Test 1: reaction to a novel object

Fifteen days after completion of individual tests, juveniles and breeders were subjected to a novel object test. For juveniles, the novel object consisted of a square wooden frame (30 × 30 × 20 cm), through which fish could pass, with an identification antenna (SQR series; TROVAN-ZEUS, Madrid, Spain) mounted in the interior. For breeders, the novel object consisted of a grey plastic cube (56.5 × 36.5 × 30 cm), supplied with an identification antenna (SQR series; TROVAN-ZEUS, Madrid, Spain) located in the bottom of the cube.

Tests started immediately after introducing the novel object in the middle of tank and lasted 24 h for both fish populations. Whether the fish passed through the novel object (juveniles) or enter into the cube (breeders) was recorded and posteriorly analysed [7,29,30]. Those fish that successfully passed through or stayed in the antenna were registered and identified.

#### 2.4.2. Test 2: risk-taking test

This test was performed on juveniles and breeders with the same behavioural criteria. The risk-taking test was conducted one month after individual tests to allow juveniles and breeders to recover. This test aimed to determine fish capacity to cross from a known area (safe zone) to an unknown area (risky zone). The safe zone was isolated from light (2 and 3 lux on the surface for juveniles and breeders, respectively) and covered with sand, to provide a comfortable and secure space for fish. On the contrary, the risky area had more illumination (15 lux on the surface for juveniles and 11 lux on the surface for breeders; OSRAM DULUX 48 and 150 W, respectively) and the bottom was devoid of sand. For juveniles, a 500 l tank was divided into two equal zones by a rigid plastic screen. A small window (5 cm high × 20 cm width) was opened at the bottom of the dividing screen, to allow fish to cross between the two areas. For breeders, the test was realized in a 16 m^3^ tank (6 m length × 3 m width × 0.9 m depth), divided into two equal areas by a wooden screen. A window (30 cm width × 15 cm tall) was placed at the base of the dividing screen with a door that could be opened to allow fish to pass from one area to another. The window for both juveniles and breeders' tanks was at the centre of a PIT tag-reading antenna (SQR series; TROVAN-ZEUS, Madrid, Spain) that was positioned to read the tag number of the fish that passed through the window to the risk zone. Additionally, two submersible black and white digital cameras (F60B/NIR580–50G model, Korea Technology Co. Ltd, supplied by Praentesis S.L., Barcelona) connected to a recorder (DVR- 0404HB model, Dahua Technology Co. Ltd, supplied by Praentesis S.L., Barcelona) were installed 10 cm below the water surface in both the safe and risky zone, to corroborate that the fish registered by the antenna had completely crossed from the safe to the risky area and not just approached the window. Constant water and aeration flows were provided during the period of the test to maintain optimal water conditions.

Before the beginning of the test, juveniles and breeders were submitted to a 24 h acclimation in the safe zone, by keeping the window closed until the beginning of the test, which started at 10.00 and lasted 24 h. Juveniles were tested in groups of 15 individuals and breeders in groups of 10 individuals to avoid inducing stress due to high stocking densities. Fish that successfully crossed from the safe zone to the risky zone were defined as ‘proactive’, while fish that did not cross were recognized as ‘reactive’ [5,7,15,29,39]. The latency time of each organism to cross from one area to another was recorded. A maximum time of 1440 min was assigned to fish that did not cross during the 24 h period of the test.

### 2.5. Cortisol, glucose and lactate analysis

A blood sample (0.5 ml) was extracted from the caudal vein of anaesthetized fish (MS-222; Argent, USA, 100 ppm) to measure cortisol, lactate and glucose concentrations. In juveniles, blood extractions were performed twice, one month before the stress coping styles tests (control), and approximately 35 min after completing all the individual tests (post-stress). In breeders, blood extraction was performed once, approximately 40 min after completing individual tests (post-stress). To avoid blood coagulation, a solution of 10 µl sodium heparin (5%, 25.000 UI; HOSPIRA) and 15 µl aprotinin (from bovine lung; 0.9% NaCl, 0.9% benzyl alcohol and 1.7 mg of protein; SIGMA) was placed inside Eppendorf tubes, while the syringes and needles used were coated with heparin. Blood samples were centrifuged (ThermoScientific centrifuge, M23i; Thermo rotor AM 2.18; 24 × 1.5 ml) at 3000*g* and 4°C for 15 min and plasma supernatant was removed and stored in triplicates at −80°C prior to analysis [26].

Cortisol was measured by a competitive conjugated binding ligand by means of a commercial ELISA kit (range of detection: 0–800 ng ml^−1^; DEMEDITEC, Kiel-Wellsee, Germany), whereas glucose and lactate were measured by means of commercial enzymatic colorimetric kits (SPINREACT, Gerona, Spain). Cortisol, glucose and lactate absorptions were read by a spectrophotometer (Infinite M-200; TECAN, Switzerland), at 23°C and 505 nm.

### 2.6. Statistical analysis

Statistical analyses were performed using SPSS Statistics 18.0 software (IBM Co., Hong Kong) and Sigmaplot 12.0 software (Systat, Inc.). Data were checked for normality by means of a Kolmogorov–Smirnov normality test. A principal components analysis (PCA), with a Kaiser–Meyer–Olkin adequacy test, Bartlett's test of sphericity and an orthogonal varimax rotation was performed in two steps: (i) individually on tests including the measurement of several variables, such as restraining, novel environment and confinement tests, in order to select the most representative variable from each test and (ii) on all variables, including cortisol, lactate and glucose concentrations, in order to select the tests that best characterized fish stress coping styles. Next, a principal component regression analysis was performed on the selected variables to generate a ‘principal component score’ (PCS) for each individual. This PCS represented the individual stress coping style behaviour of each fish for each of the most representative selected variables. Subsequently, a general linear multivariate model analysis (GLMM) and Kolmogorov–Smirnov test (KS-test), for independent populations, were performed to examine the inter-individual consistency between the group of juveniles and breeders for the selected tests resulting from the PCA. In the group tests, the PCS that was assigned to fish that successfully crossed in the risk-taking test was compared to the PCS of fish that did not cross, by means of Student *t*-test. Such comparisons were made in order to support the effectiveness of the risk-taking test to discriminate proactive fish from reactive fish within the selected tests. While, the consistency was checked by comparing the proportion of fish (juvenile and breeders) that crossed and those that did not cross in the risk-taking test by a *χ*^2^ test. To check if the latency time to cross into the risk area in the risk-taking test was correlated with the latency time in the new environment and confinement tests, a Pearson correlation analysis was performed. Lastly, the coefficient of variation (CV % = s.d./mean × 100) was averaged for each test and represented the inter-individual variability of sole. For each parameter of the selected coping style tests, fish were separated into four quartiles that represented low to high activity categories, by an Ntiles rank analysis and the mean glucocorticoids production was compared by ANOVAs between the low and high activity groups, for initial and post-stress levels in juveniles and for post-stress levels in breeders. Across-context correlations between the PCS and fish weight, length, and cortisol, glucose and lactate concentrations were performed using intraclass correlation coefficient analysis (ICC). When various correlations were performed on the same dataset, the procedure of Benjamini & Hochberg was implemented, using the R Package (R Development Core Team, https://www.r-project.org), to correct probabilities and control Type 1 error [40,41]. A *p*-value < 0.05 was established as statistically significant for all tests realized.

## 3. Results

### 3.1. Senegalese sole behavioural responses in individual tests

Tests induced different responses in Senegalese sole juveniles and breeders. In the restraining test, behavioural responses of both juveniles and breeders ranged from fish that did not move and remained static in the net to fish that immediately attempted to escape with a high level of activity (escape attempts and time of activity) during the 90 s. Juveniles showed different behavioural motivation than breeders. Indeed, juveniles showed significantly higher activity (*p* < 0.016), escape attempts (*p* < 0.001) and less variability (CV NetActA = 57.4%; CV NetEscA = 65.8%) outside the water, while breeders presented significant higher activity (*p* < 0.001), escape attempts (*p* < 0.001) and less variability (CV NetActW = 94.9%; NetEscW = 133.9%) inside the water (tables 1 and 2). In both the new environment and confinement tests, behavioural responses ranged from fish that did not move and appeared to freeze on being introduced into the container to fish that immediately moved (low latency time) with a high level of activity during the 300 s and consequentially the variation was high ranging from CV = 88.6% (juvenile NewLat) to 167.2% (juvenile NewAct) (tables 1 and 2). Moreover, the GLMM showed high inter-individual consistency, because latency time and total activity time in new environment and confinement tests of juveniles and breeders were not significantly different (*F*_116_ = 2.00, *p* = 0.159 and *F*_116_ = 0.118, *p* = 0.732; *F*_116_ = 1.00, *p* = 0.319 and *F*_116_ = 0.066, *p* = 0.798, respectively) and showed similar distributions (KS-test, NewLat *p* = 0.785, NewAct *p* = 0.430, ConLat *p* = 0.110 and ConAct *p* = 0.158). Juveniles showed significant correlations between NetActA and ConAct (*r* = 0.491; adjusted *p* = 0.003), between NetEscA and ConLat (*r* = 0.439; adjusted *p* = 0.0106), between NetEscA and ConAct (*r* = 0.556; adjusted *p* = 0.0003). In breeders, the highest correlations were noted between NetActW and NewLat (*r* = 0.455; adjusted *p* = 0.0106) and between NetActW and NewAct (*r* = 0.465; adjusted *p* *=* 0.009). These correlations suggested that more active individuals in the restraining test were also more active in the new environment test, showed lower latency to start exploration and indicated intra-individual consistency across contexts. The latency time in the new environment and confinement tests of juveniles and breeders appeared to adjust to a bimodal distribution. However, this was related to assigning 300 s to individuals that did not move during the test as the second mode consisted predominantly of these inactive fish. The true distribution cannot be known as responses after 300 s were not collected. A highly positively skewed or bimodal distribution would be expected considering the number of fish in the second mode. No significant correlations (adjusted *p* > 0.05) were observed between the variables from the restraining, new environment or confinement tests with cortisol, glucose or lactate levels, weight and length, neither for juveniles nor for breeders.

**Table 1.** General results for individual coping style tests of Senegalese sole juveniles (*n* = 61). The CV of latencies in new environment and in confinement were calculated with the total averaged mean. Superscript letters reveal significant differences (analysed by Student *t*-test).

coping style tests	variables	type of distribution	mean ± s.d. (1st mode)	mean ± s.d. (2nd mode)	min	max	coef. variance (%)
restraining	total activity inside water (s)	skew	14.80 ± 14.8^A^	—	0	76	100
	escape attempts inside water	skew positive	2.9 ± 4.1^A^	—	0	16	141.4
	total activity outside water (s)	normal	10.1 ± 5.8^B^	—	1	23	57.4
	escape attempts outside water	normal	27.5 ± 18.1^B^	—	0	67	65.8
new environment	latency time in new environment (s)	bimodal	28.32 ± 6.6	295.5 ± 3.1	2	300	88.6
	total activity in new environment (s)	skew positive	11.6 ± 19.4	—	0	157	167.2
confinement	latency time in confinement (s)	bimodal	31.2 ± 7.1	294.6 ± 3.9	1	300	105.2
	total activity in confinement (s)	skew positive	31.3 ± 42.5	—	0	70	135.8
	total opercula openings	skew positive	49.6 ± 47.2	—	1	154	95.2
flipping fish over	time to recover normal position (s)	skew positive	115.6 ± 110.2	—	1	180	95.3
novel object	total fish that crossed the novel object	skew	71.6 ± 97.1	—	0	428	135.6
blood hormones (control)	cortisol (ng ml^−1^)	normal	32.1 ± 25.1	—	8.3	132.7	78.2
	lactate (mmol l^−1^)	normal	19.4 ± 5.1	—	11.5	30.1	26.3
	glucose (mmol l^−1^)	normal	4.0 ± 1.7	—	1.6	12.2	42.5
blood hormones (post-stress)	cortisol (ng ml^−1^)	skew positive	79.6 ± 64.8	—	12.9	265.6	81.1
	lactate (mmol l^−1^)	normal	26.8 ± 5.6	—	15.3	47.1	20.9
	glucose (mmol l^−1^)	normal	6.2 ± 3.1	—	2.8	20.1	50

**Table 2.** General results for individual coping style tests of Senegalese sole breeders (*n* = 59). The CV of latencies in new environment and in confinement was calculated with the total averaged mean. Superscript letters reveal significant differences (analysed by Student *t*-test).

coping style tests	variables	type of distribution	mean ± s.d. (1st mode)	mean ± s.d. (2nd mode)	min	max	coef. variance (%)
restraining	total activity inside water (s)	skew positive	17.80 ± 16.9^A^	—	0	73	94.9
	escape attempts inside water	skew positive	5.30 ± 7.18^A^	—	0	31	133.9
	total activity outside water (s)	skew positive	3.40 ± 6.13^B^	—	0	26	180.2
	escape attempts outside water	skew positive	3.00 ± 6.4^B^	—	0	30	200
new environment	latency time in new environment (s)	bimodal	28.44 ± 6.05	291.72 ± 6.01	0	300	117.8
	total activity in new environment (s)	skew positive	26.10 ± 35.8	—	0	166	137.6
confinement	latency time in confinement (s)	bimodal	22.55 ± 3.95	291.74 ± 5.75	0	300	115.7
	total activity in confinement (s)	skew positive	24.20 ± 36.3	—	0	184	150
flipping fish over	time to recover normal position (s)	skew negative	143.70 ± 65.1	—	1	180	45.3
novel object	total fish that crossed the novel object	—	0		0	0	
anaesthesia	light sedation (s)	normal	66.6 ± 29.9	—	14	160	44.9
	total sedation (s)	normal	146.70 ± 81.15	—	4	559	55.3
	deep sedation (s)	normal	251.40 ± 145.0	—	68	980	57.5
blood hormones (post-stress)	cortisol (ng ml^−1^)	skew	20.60 ± 55.17	—	0.1	318	267.4
	glucose (mmol l^−1^)	normal	4.75 ± 2.70	—	0.91	10.36	55.4
	lactate (mmol l^−1^)	normal	6.60 ± 6.40	—	0.05	25.65	96.4

In the opercula openings variable (table 1), juveniles averaged 49.6 ± 47.2 movements during 1 min and presented considerable variation (CV = 95.2%). However, this test was difficult to assess in this flatfish species because of the position of gill. In the flip-over test (tables 1 and 2), only 14 juveniles from a total of 61 recovered their normal position after an average time of 60.4 ± 48.65 s (the average time for all juveniles (*n* = 61) was 115.6 ± 110.2 s and CV = 95.3%), while 17 breeders of 59 recovered their normal position after an average time of 47.1 ± 39.3 s and in comparison to other tests variation in individual responses was low (the average time for all breeders (*n* = 59) was 143.7 ± 65.1 s and CV = 45.3%). Fish weight appeared to be an issue for this test, because the heaviest fish showed more difficulties in flipping over than lighter fish, therefore this test was not considered a reliable test to characterize stress coping styles in Senegalese sole. In the anaesthesia test, breeders reached the light sedation level after 66.6 ± 29.9 s, the total sedation level after 146.7 ± 81.1 s and the deep sedation level after 251.8 ± 145.1 s. This test did not show much behavioural variability (CV light sedation = 44.9, CV total sedation = 55.3; CV deep sedation = 57.3%) and, therefore, may not express a large variation in relation to stress coping styles (table 2). Lastly, the juveniles' post-stress cortisol concentration (79.6 ± 64.8 ng ml^−1^) was significantly higher (*p* < 0.001) than their basal level (32.1 ± 25.1 ng ml^−1^), but glucose and lactate concentrations were not statistically different (*p* > 0.05) and variation was relatively low. In breeders, the post-stress levels were 20.60 ± 55.17 ng ml^−1^ for cortisol, 4.70 ± 2.67 mmol l^−1^ for glucose and 6.63 ± 6.39 mmol l^−1^ for lactate (tables 1 and 2). Breeders' individual response variation was higher than in juveniles, especially for cortisol (CV = 267.4%).

**Figure 1.**
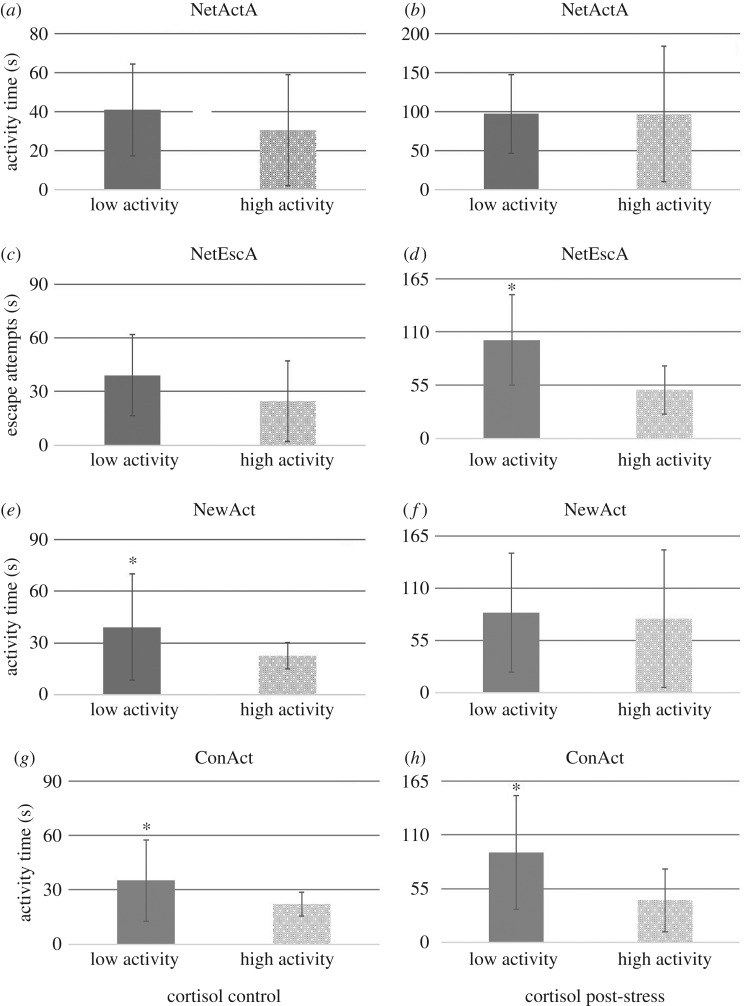
Differences between juveniles with low (lower quartile of Ntyles analysis) and high (higher quartile of Ntyles analysis) activity levels and cortisol concentration (control and post-stress) in the restraining (*a*–*d*), new environment (*e,f*) and confinement (*g,h*) tests. Asterisk indicates significant differences.

**Figure 2.**
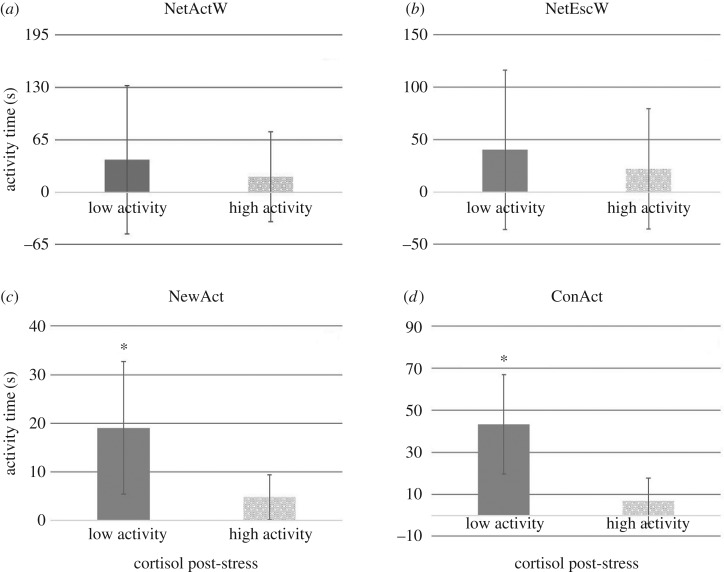
Differences between breeders with low (lower quartile of Ntyles analysis) and high (higher quartile of Ntyles analysis) activity levels and cortisol concentration (post-stress) in the restraining (*a*,*b*), new environment (*c*) and confinement (*d*) tests. Asterisk indicates significant differences.

Further, the fish from the high activity quartile (ranked by an Ntiles-analysis for measures of activity, escape attempts in the net and activity in the new environment and confinement) had significantly lower cortisol than fish in the low activity quartile in juveniles (figure 1) for total activity in new environment (control cortisol *F*_1, 39_ = 4.10, *p* = 0.050), escapes attempts outside water (post-stress cortisol *F*_1, 28_ = 6.16, *p* = 0.019) and total activity in confinement (control cortisol *F*_1, 33_ = 4.75, *p* = 0.038 and post-stress cortisol *F*_1, 33_ = 6.58, *p* = 0.015) and in breeders (figure 2) also for total activity in new environment (post-stress cortisol *F*_1, 28_ = 4.26, *p* = 0.048) and total activity in confinement (post-stress cortisol *F*_1, 29_ = 6.89, *p* = 0.014). This indicated that selected tests were in line with the differences in cortisol production observed between proactive and reactive fish in both juveniles and breeders.

### 3.2. Behavioural responses of sole in the group tests

Fifty-nine juveniles of the 61 passed through the novel object and 2 fish never passed, while none of the 59 breeders entered or even approached the identification antenna in the novel object (tables 1 and 2). In addition, a high variability was observed in the number of juveniles passing through the novel object (CV = 135.6%).

**Figure 3.**
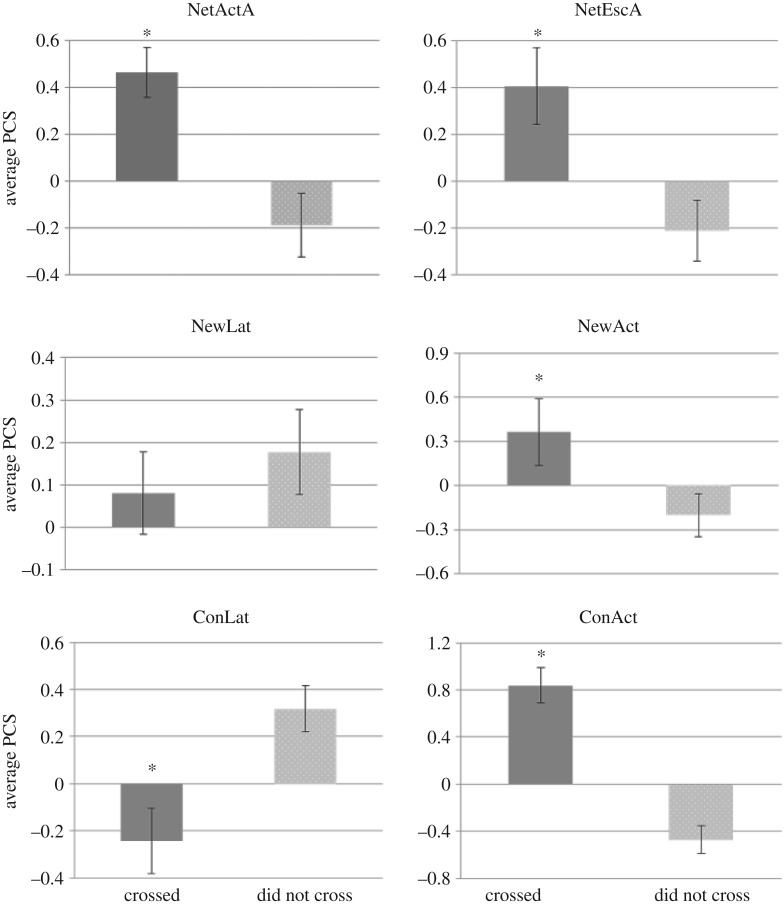
Principal component scores (PCS) of juveniles that successfully crossed versus those that did not cross in the risk-taking test, for NetActA, net activity in air; NetEscA, net escape in air; NewLat, latency in new environment; NewAct, activity in new environment; ConLat, latency in confinement and ConAct, activity in confinement. Asterisk indicates statistical differences.

**Figure 4.**
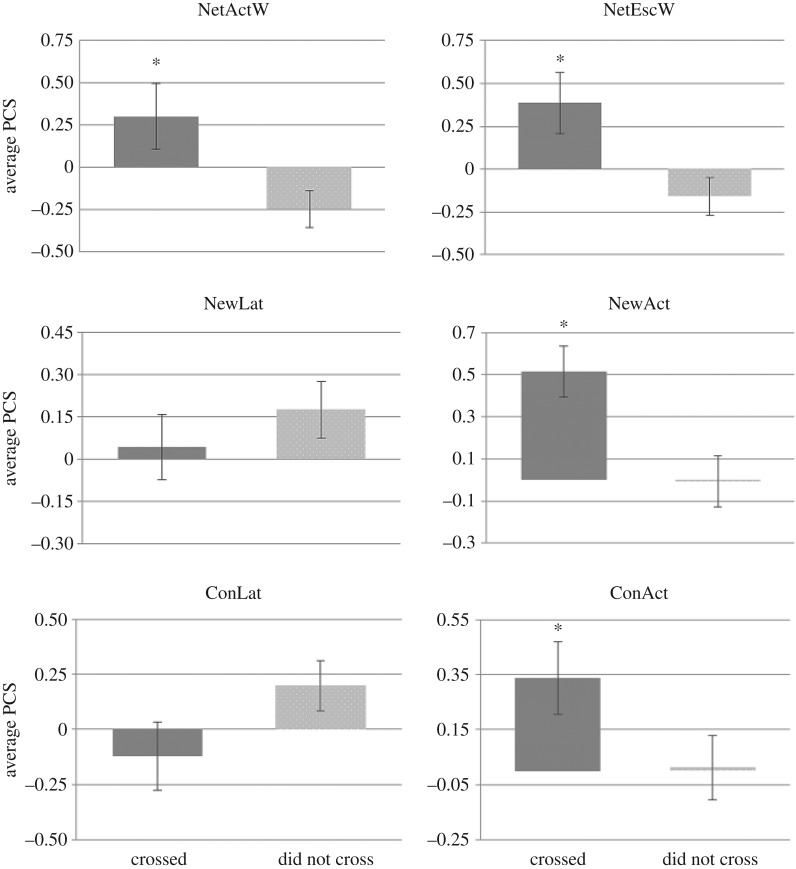
Principal component scores (PCS) of breeders that successfully crossed versus those that did not cross in the risk-taking test, for NetActW, net activity in water; NetEscW, net escapes in water; NewLat, latency in new environment; NewAct, activity in new environment; ConLat, latency in confinement and ConAct, activity in confinement. Asterisk indicates statistical differences.

In the risk-taking test, 22 juveniles of 61 crossed from the safe area to the risky area (36%) in an average time of 337.5 ± 177.1 min after starting the test (table 3), while 17 breeders of 59 (29%) crossed in an average time of 414.1 ± 187 min after starting the test (table 4). The observed proportion of juveniles that successfully crossed was consistent with the proportion of breeders that crossed (*χ*^2^ = 0.719, d.f. = 1, *p* = 0.396). The PCS of juveniles that successfully crossed showed significantly higher activity levels (NetActA, *t* = 5.94, d.f. = 59, *p* = 0.001; NetEscA, *t* = 7.23, d.f. = 59, *p* = 0.008; ConAct, *t* = 6.98, d.f. = 59, *p* = 0.001 and NewAct, *t* = 5.50, d.f. = 59, *p* = 0.024) and significantly lower latencies (ConLat; *t* = 3.12, d.f. = 59, *p* < 0.001) than juveniles that did not cross (figure 3). Besides, PCS of breeders that successfully crossed showed significantly higher activity levels (NetActW, *t* = 3.63, d.f. = 57, *p* = 0.012; NetEscW, *t* = 3.62, d.f. = 57, *p* = 0.011; NewAct, *t* = 3.28, d.f. = 57, *p* = 0.014 and ConAct, *t* = 1.28, d.f. = 57, *p* = 0.042) than fish that did not cross (figure 4). Moreover, both juveniles and breeders that successfully crossed in the risk-taking test showed lower post-stress cortisol levels than fish that did not cross (tables 3 and 4). This result suggests that proactive (also named ‘bold’ fish)—assumed to be those that successfully crossed in the risk-taking test—were confirmed to present higher activity in the restraining, new environment and confinement tests than reactive (also named ‘shy’ fish)—assumed to be those that did not cross in the risk-taking test. Lastly, the latency time to cross was not correlated with the latency in new environment (*r* *=* 0.304, adjusted *p* = 0.932) and neither with the latency in confinement (*r* *=* 0.025, adjusted *p* = 0.788).

**Table 3.** Average values of coping style variables measured for juveniles that crossed (*n* = 22) versus those that did not cross (*n* = 39) in the risk-taking test. Superscript letters indicates significant differences.

coping style tests	variable	crossed	did not cross
restraining	total activity inside the water (s)	20.5 ± 3.8^a^	11.5 ± 1.8^b^
	escape attempts inside the water	4.1 ± 1.1	2.3 ± 0.5
	total activity outside the water (s)	11.5 ± 1.2^a^	9.2 ± 0.9^b^
	escape attempts outside the water	39.7 ± 3.6^a^	20.6 ± 2.3^b^
new environment	latency time in new environment (s)	142.8 ± 27.7	162.5 ± 22.9
	total activity in new environment (s)	17.4 ± 5.2^a^	8.2 ± 24^b^
confinement	latency time in confinement (s)	50.3 ± 16.8^a^	168.9 ± 22.1^b^
	total activity in confinement (s)	61.1 ± 10.3^a^	14.6 ± 4.4^b^
	total opercula openings	54 ± 10.6	47.1 ± 7.3
flipping fish over	time to recover normal position (s)	125.6 ± 24.3	109.5 ± 17.4
novel object	total fish that crossed the novel object	84.9 ± 27.1	64.2 ± 12.2
blood hormones (control)	cortisol (ng ml^−1^)	21.1 ± 1.8^a^	38.3 ± 4.6^b^
	lactate (mmol l^−1^)	20.1 ± 1.1	19.1 ± 0.8
	glucose (mmol l^−1^)	3.7 ± 0.2	4.1 ± 0.2
blood hormones (post-stress)	cortisol (ng ml^−1^)	41.8 ± 6.2^a^	100.9 ± 11.1^b^
	lactate (mmol l^−1^)	25.9 ± 1.1	27.3 ± 0.9
	glucose (mmol l^−1^)	5.8 ± 0.7	6.4 ± 0.4

**Table 4.** Average values of coping style variables for breeders that crossed (*n* = 17) versus those that did not cross (*n* = 42). Superscript letters indicates significant differences.

coping style tests	variables evaluated	crossed	did not cross
restraining	total activity inside the water (s)	21.5 ± 3.8^a^	16.3 ± 2.6^b^
	escape attempts inside the water	6.6 ± 1.8	4.8 ± 1.1
	total activity outside the water (s)	5.5 ± 1.9	2.6 ± 0.8
	escape attempts outside the water	4.12 ± 1.7	2.5 ± 0.9
new environment	latency time in new environment (s)	100.5 ± 30.4	113.2 ± 20.3
	total activity in new environment (s)	35.2 ± 8.3^a^	22.4 ± 5.6^b^
confinement	latency time in confinement (s)	113.2 ± 31.7	108 ± 19.5
	total activity in confinement (s)	19.5 ± 8.2	26.1 ± 5.8
flipping fish over	time to recover normal position (s)	116.1 ± 17.7^b^	155 ± 9.1^a^
anaesthesia	light sedation (s)	64.6 ± 8.2	67.3 ± 4.4
	total sedation (s)	138.6 ± 16.1	150 ± 13.4
	deep sedation (s)	281.1 ± 50.2	239.3 ± 17.2
blood hormones (post-stress)	cortisol (ng ml^−1^)	3.3 ± 1.1^b^	27.6 ± 9.9^a^
	glucose (mmol l^−1^)	5.0 ± 0.8	4.6 ± 0.4
	lactate (mmol l^−1^)	6.1 ± 1.1	6.8 ± 1.1

### 3.3. Coping style tests selection

**Figure 5.**
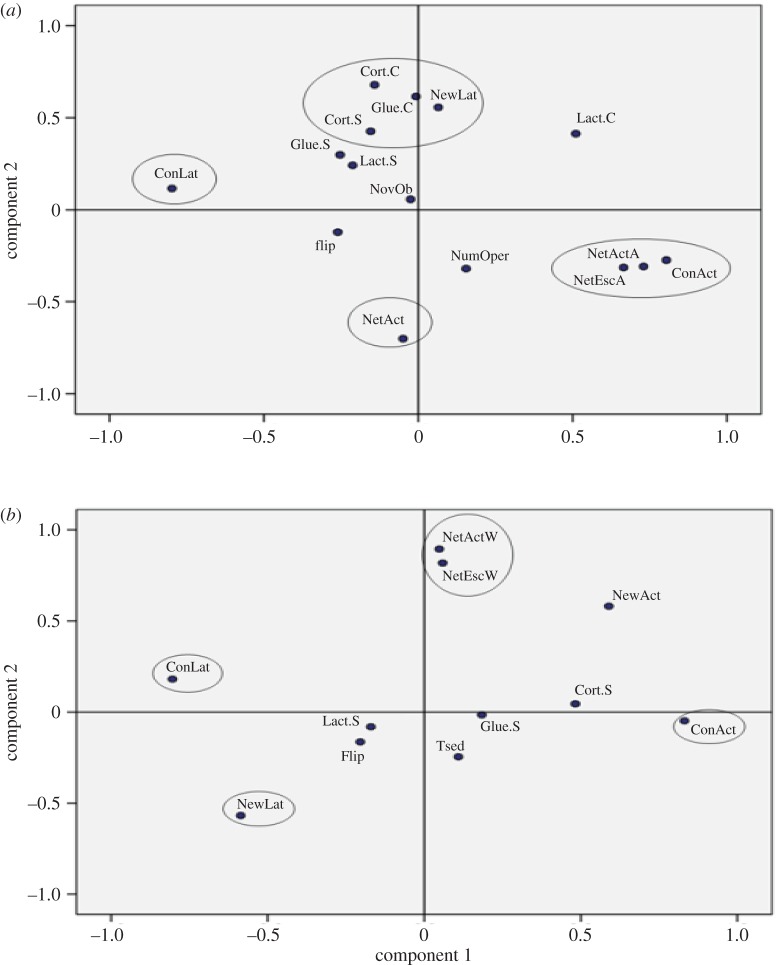
Component matrix diagrams of variables selected (in circled) to characterize stress coping style of Senegalese sole juveniles (*a*) and breeders (*b*).

The efficacy of the PCA was confirmed by the significance of the results of Kaiser–Meyer–Olkin test and Bartlett's test of sphericity results (KMO = 0.574 and 0.588; *χ*^2^ = 229.80, d.f. = 105, *p* < 0.001 and *χ*^2^ = 102.43, d.f. = 55, *p* < 0.001, for juveniles and breeders, respectively). From all behavioural variables evaluated in the PCA, three main tests resulted appropriate to characterize Senegalese sole juveniles' and breeders' stress coping styles: (i) restraining test, (ii) novel environment test, and (iii) confinement test (figure 5*a*,*b*). These three selected tests described more than 70% of the total variance for juveniles' behavioural differences and 74% for breeders in the principal component matrix, when represented by two principal components (PC1 and PC2). Those tests also had the highest communalities and eigenvalues (tables 5 and 6). Component one of PCA showed high context correlations between NewLat-NewAct and ConLat-ConAct (new environment and confinement variables) for juveniles and breeders. In component two, the highest context correlations observed for juveniles corresponded to variables NetActA-NetEscA (netting the fish outside the water) and cortisol concentration before and after performing the stress coping style tests, while in breeders the highest correlations corresponded to variables NetActW-NetEscW (netting the fish inside the water) and cortisol levels (tables 5 and 6).

**Table 5.** PCA scores, eigenvalues and percentage of variance explained of the different coping style tests performed in Senegalese sole juveniles. Variables marked in italics were selected.

coping style tests	variable	component matrix score PC1	component matrix score PC2	communality	eigenvalue	% variance explained
restraining	total activity inside the water (s)^a^		0.253			
	escape attempts inside the water^a^		−0.422			
	*total activity outside the water* (*s*)	* *	*0*.*744*	*0*.*628*	*1.871*	*12.47*
	*escape attempts outside the water*	* *	*0*.*708*	*0*.*641*	*1.641*	*10.94*
new environment	*latency time in new environment* (*s*)	*0*.*665*	* *	*0*.*514*	*1.135*	*7.565*
	*total activity in new environment* (*s*)	−*0*.*562*	* *	*0*.*494*	*1.107*	*7.379*
confinement	*latency time in confinement* (*s*)	*0*.*679*	* *	*0*.*650*	*1.351*	*9.01*
	*total activity in confinement* (*s*)	−*0*.*787*	* *	*0*.*721*	*3.444*	*22.96*
	total opercula openings^a^	−0.327				
flipping fish over	time to recover normal position (s)^a^	0.117				
novel object	total fish that crossed the novel object^a^	0.057				
blood hormones (control)	cortisol (ng ml^−1^)^a^		0.678	0.556	0.981	
	lactate (mmol l^−1^)^a^		0.413			
	glucose (mmol l^−1^)^a^		0.141			
blood hormones (post-stress)	cortisol (ng ml)^a^		0.426	0.480	0.810	
	lactate (mmol l^−1^)^a^		0.242			
	glucose (mmol l^−1^)^a^		0.299			

^a^Not considered as eigenvalue was less than 1.

**Table 6.** PCA scores, eigenvalues and percentage of variance explained of the different coping style tests performed in Senegalese sole breeders. Variables marked in italics were selected.

coping style tests	variable	component matrix score PC1	component matrix score PC2	communality	eigenvalue	% variance explained
restraining	*total activity inside the water* (*s*)		*0*.*735*	*0*.*802*	*2*.*946*	*10*.*96*
	escape attempts inside the water^a^		0.682	0.672	0.876	
	total activity outside the water (s)^a^		−0.482			
	escape attempts outside the water		0.344			
new environment	*latency time in new environment* (*s*)	*−0*.*815*		*0*.*665*	*1*.*298*	*15*.*43*
	*total activity in new environment* (*s*)	*0*.*827*		*0*.*686*	*1*.*698*	*26*.*77*
confinement	*latency time in confinement* (*s*)	*−0*.*692*		*0*.*693*	*1*.*006*	*9*.*14*
	*total activity in confinement* (*s*)	*0*.*785*		*0*.*680*	*1*.*206*	*11*.*81*
flipping fish over	time to recover normal position (s)^a^	0.261				
anaesthesia	light sedation (s)^a^	0.206				
	total sedation (s)^a^	0.083				
	deep sedation (s)^a^	0.101				
blood hormones (post-stress)	*cortisol (ng ml^−1^)*		*0*.*485*	0.465	0.764	—
	glucose (mmol l^−1^)^a^		−0.181			
	lactate (mmol l^−1^)^a^		0.126			

^a^Not considered as eigenvalue was less than 1.

## 4. Discussion

### 4.1. Characterization of stress coping style and test selection for operational behavioural screening test

Three tests were demonstrated to enable the characterization of stress coping style of Senegalese sole juveniles and breeders: (i) restraining test, (ii) new environment test and (iii) confinement test. The restraining test, which measured the ability of fish to respond to invasive and aversive situations, induced two divergent behavioural reactions in juveniles and breeders characterized by high and low: activity and escapes attempts. Thus, proactive juveniles and breeders were actively attempting to escape, while reactive individuals did not make escape attempts or move in the net. Therefore, confirming the test was reliable to sort proactive and reactive behaviours [25–29]. Similarly, the new environment and confinement tests induced high amplitude of individual behavioural variability in the latency time and total activity time responses that were consistent with coping styles. Thus, proactive juveniles and breeders tended to resume activity earlier and showed higher activity than reactive individuals, suggesting a higher-explorative behaviour and reactivity to stressful situations. These behavioural observations were similar to those reported in bluegill sunfish *Lepomis macrochirus* [5,42], gilthead seabream [28] and swordtail *Xiphophorus birchmanni* [43], with proactive fish displaying higher activity and explorative behaviour in comparison to reactive fish. Other individual tests, flip over, anaesthesia and opercula movements were not selected as appropriate tests. The principal reason to reject these tests was low variance across the population and lower correlations with other tests. Low variance in opercula movements for Senegalese sole could be related to the observation that common sole (*S. solea*), a closely related species, reduce ventilation rates when startled [44]. Therefore, in a similar way, Senegalese sole in this study may have reduced ventilation rates in the tests and thus reduced variance between behavioural phenotypes. In addition, there were some technical issues such as observing slight opercula movements and in the flip-over test, results appeared to be compromised by fish size, particularly for breeders. These considerations indicate that opercula movements, anaesthesia and the flip-over test may not be appropriate to determine the stress coping style of sole.

### 4.2. Latency in new environment and confinement tests

The latency until first activity in new environment and confinement adjusted to a bimodal frequency distribution in both Senegalese sole juveniles and breeders, because some fish resumed activity almost immediately after being introduced into the tests and others did not move during the entire 300 s (5 min) of the test. These soles that did not move were recorded as having a latency to first activity of 300 s. It should be understood that it was not known when these fish that did not move would have moved and that the bimodality may actually represent a highly positively skewed population (if fish moved at random points after 300 s) or a true bimodal situation (if the first movement of these fish was grouped around a particular time point). This raises the question of the best way to treat the data, here as in other publications [29] the fish that did not move were scored with a maximum time of the test and the data were analysed as continuous. An alternative would be to record a binomial response i.e. fish that moved and fish that did not move. There are arguments and advantages for and against the two approaches. Both approaches and analysis of the data from this study (binomial approach and analysis not included in this study) gave similar results and the same conclusions. The use of continuous data was preferred as it provided a more variable response and a more robust analysis (more significance and variability) and enables the analysis to determine a stress coping style response rather than scoring organisms as proactive (active in the tests) or reactive (inactive in the tests). Despite of these considerations the bimodality appeared to represent the two extremes of personalities and possibly explained how fast proactive fish acclimate to a new environment or how fast they react to escape from confinement situation. This assumption agrees to the concept proposed by Fox *et al*. [45] and Budaev & Brown [46]. These authors indicated that bimodality in behavioural responses to coping style tests is a common factor in several fish species, because individuals usually tend to form clusters of similar traits rather than continuously distributed traits or dimensions.

### 4.3. Intra-individual consistency across tests (context)

Each test found intra-individual consistency in the behavioural responses across context within each group, juveniles and breeders. There were high context correlations (PC1 of PCA) between the new environment and confinement tests indicating a high degree of intra-individual consistency across these two context. These two tests, new environment and confinement, also exhibited a degree of correlation with the restraining test for juveniles and breeders (*r* = 0.439 to *r* = 0.556) indicating intra-individual consistency across these contexts.

The risk-taking test has been widely accepted as a test that discriminated proactive from reactive individuals in other fish species, such as bluegill sunfish [5], common carp [15] and gilthead seabream [28]. In this study, the risk-taking test was also successfully used to both (i) discriminate proactive from reactive individuals in both groups of fish (juveniles and breeders) and (ii) showed intra-individual consistency to validate the identification of proactive and reactive fish identified the three selected tests (restraining, new environment and confinement). Indeed, the tests revealed that those juveniles and breeders with higher activity in the three selected tests were those with higher predisposition to take risk (e.g. cross from safe to risk zone). However, the inadequacy of the risk test for aquaculture, in comparison of the three individual tests selected, is the long time period required for it to be performed.

Physiological response, particularly cortisol, has been identified and accepted as a characteristic of proactive and reactive organisms, with proactive organisms exhibiting a low cortisol response and reactive organisms exhibiting a high cortisol response to a stressor [27,28,33]. Perhaps contradicting the cross-context correlations (across the three selected tests and the risk test), the three tests exhibited poor correlations with the physiological parameters indicating that the behavioural responses were not consistent with physiological responses. However, it should be noted that even though correlations were poor with physiological parameters, the fish that were identified as proactive (high activity quartile) with the three tests, restraining, new environment and confinement had significantly lower levels of cortisol compared to fish identified as reactive (low activity quartile), which suggests there was a degree of cross-context intra-individual consistency and that these tests identify stress coping styles in agreement with the physiological dimension to give a further validation of the three selected tests. Moreover, control and post-stress levels of cortisol in juveniles and post-stress levels of cortisol in breeders were consistent with those reported for other fish species, such as gilthead seabream [28] and sea bass (*Dicentrarchus labrax*) [47]. These results are in agreement with other studies that established cortisol as stress coping style predictor and confirmed the lower HPI axis reactivity to stress in proactive fish than in reactive fish [27,28,33].

### 4.4. Inter-individual consistency across groups

The behavioural profiles of juveniles and breeders were similar between two selected tests, the new environment and confinement, because the general linear model and the Kolmogorov–Smirnov tests showed no differences in the inter-individual variation between the two groups. In addition, the *χ*^2^-test showed no statistical differences between the proportion of juveniles and breeders crossing from the safe to risky area in the risk taking. These results demonstrated inter-individual consistency across context (juveniles compared to breeders) and confirmed the existence of behavioural syndromes in different situations (e.g. high activity and escape attempts in juveniles and breeders resulted in low latency and high total activity) in this fish species [3,48]. Correlations or consistency between behaviours in different contexts or situations may have important implications in animals, because they might generate trade-offs between different behaviours [3,31,48]. Therefore, it is possible that this consistency between juveniles and breeders relies on the fact that behaviours are linked because they are both governed by a common physiological mechanism.

### 4.5. Use of component analysis to identify tests and behavioural syndromes

It is worth noting that the PCA analysis represented a practical statistical method to reduce variables and characterized coping styles of Senegalese sole juveniles and breeders. The three selected tests presented the highest weight in the first two components (PC1 and PC2) of the PCA and explained the highest variance. Studies using PCA to characterize animal behaviour have been performed not only in fish [28,49,50] but also in other vertebrates such as birds [51] and mammals [52]. In relation to the two main extracted PCA components, it seemed that PC1 represented the fish ‘activity-exploration behaviour’ due to the high PCA context correlations observed between new environment activity–latency and confinement activity–latency, while PC2 characterized Senegalese sole ‘fearfulness-reactivity behaviour’ because of the high PCA context correlations between activity in the restraining test in air and cortisol concentrations (control and post-stress) for juveniles and between activity in the restraining test in water and cortisol concentrations (post-stress) for breeders. The ‘activity-exploration behaviour’ was defined by the high activity and explorative behaviour showed by juveniles and breeders when submitted to new environments or into a confinement situation, which closely resembles curiosity, impulsiveness or reactiveness to the presence–absence of conspecifics, space and sociability. The ‘fearfulness-reactivity behaviour’ was interpreted as the reaction of fish in relation to an aversive situation that resembles fear, stimulation and anxiety. The interpretation of components reflecting behavioural traits or syndromes, as in this study, have been previously described in others fish species, such as guppy (*Poecilia reticulata*) [39], Nile tilapia [53] and brown trout [54], by means of similar tests and statistical analysis to those used in this study to characterize fish stress coping styles.

However, although similar behavioural axes were found across different fish species, different behavioural tests have been plotted in the PCA to represent the behavioural axes. In particular, the confinement test has been both included in ‘activity-exploration behaviour’ axis in correlation with the new environment test (this study; [39]) and in ‘fearfulness-reactivity behaviour’ axis in correlation with the restraining test [55]. This difference may be due to methodological differences in executing the confinement test or intrinsic biological differences between species. The confinement assay has been considered as a fearful test because it induces differences in cortisol, a factor that is strongly linked to behaviours indicating fearfulness [30]. However, the duration in confinement was considerably higher in studies that utilized confinement to discriminate for stress coping styles (30 min at least, [17,32,33,56]) compared with this study (5 min). Moreover, these studies [17,25,30,32,33,55,56] were performed on round fishes, whereas in a flatfish species such as Senegalese sole, the perception of this test was probably different. Placing a fish in confinement where swimming is difficult or not possible would have different effects on a round fish that is constantly swimming compared with a flatfish that leads a largely stationary life on the bottom and differences in the species reaction to confinement would be expected and could be tested by looking at cortisol and other stress indicators. Therefore, methodological and biological differences identified between previous studies and this study with sole may explain why the confinement test was correlated with the new environment test in the PC1 and not with the restraining test. Nonetheless, it would be interesting to perform further studies combining these three tests on other round fish and flatfish species, in order (i) to confirm the associations obtained in each of the generated components, (ii) to examine stress response to the tests and (iii) to get a more solid basis for the establishment of the axis of personality proposed.

### 4.6. Advantages of selected tests for the aquaculture industry (operational behavioural screening test)

In aquaculture, the operational success of culturing fish depends, among other things, on providing optimal rearing conditions to improve fish welfare. Understanding fish coping styles may minimize stress responses, resulting in increased production, survival, growth, resistance to diseases and, ultimately, improved reproduction. Further, the importance to understand why animals behave as they do should not be neglected, because it is through their behaviour that animals interact and cope with the environment [57,58]. The effect of external stimuli of the fish held in captivity (i.e. human presence, fish manipulation, feeding, etc.) might result in different behavioural responses, because animals react differently to the same stimulus as a function of their different personalities or stress coping styles [20,56–58]. Therefore, the selection of certain personality traits might improve the efficiency of aquaculture productivity. The three coping style tests proposed in this study might prove attractive and feasible for sole farmers, because: they are easy to perform, many individuals can be tested in a short amount of time, are relatively inexpensive and do not need modifications of the rearing systems. These tests are individually based and can be performed quickly on a low or high number of fish in comparison to other tests. Moreover, these tests could be easily achieved and interpreted by farmers without experience in fish behaviour. In addition, the selected tests (i) confirmed two personality axes that are well accepted in literature, being the fearfulness-reactivity behaviour and the activity-exploration behaviour and both axes exhibited significant differences from each other, (ii) the selected tests exhibited intra- and inter-individual consistency, (iii) the tests were representative of the physiological stress coping style response as the proactive fish that were identified had significantly lower cortisol levels than reactive fish, and (iv) the selected tests were validated by the risk-taking test for identifying proactive and reactive individuals. Incorporating suitable and reliable tests to rearing protocols to characterize stress coping style of Senegalese sole juveniles and breeders may generate valuable information to fish farmers in order to detect, for example, how efficiently fish are able to adapt to their environment and captive rearing conditions and husbandry.

## 5. Conclusion

This study confirmed the existence of proactive and reactive behaviours in Senegalese sole breeders and proposed three individual stress coping style tests that identify individual behavioural differences in Senegalese sole juveniles and breeders, kept under aquaculture rearing conditions. Moreover, the three selected tests might be used as an operational screening method (OBST) in the aquaculture industry to select proactive and reactive individuals. Understanding Senegalese sole stress coping styles may benefit fish farmers to improve fish welfare and production systems, which ultimately may result in the establishment of selection-based breeding programmes, to improve domestication and produce fingerlings with specific behavioural traits. Finally, more studies should be performed in order to increase the knowledge on Senegalese sole coping styles in relation to growth, food conversion, disease resistance, reproductive success, fitness, repeatability and dominance.

## Supplementary Material

Ibarra-Zatarain et al., 2016 Senegalese sole stress coping style data.

## Data accessibility

The datasets supporting this article have been uploaded at Dryad [61]: http://dx.doi.org/10.5061/dryad.4f3f6.

## Glossary

OBST: operational behavioural screening tests
*Restraining test*
NetActW: total activity inside water
NetActA: total activity outside water
NetEscW: escape attempts inside water
NetEscA: escape attempts outside water
*New environment test*
NewLat: first activity time in new environment
NewAct: total activity time in new environment
*Confinement test*
ConLat: first activity time in the confinement
ConAct: total activity time in the confinement
NumOper: total opercula openings
*Flipping fish over test*
Flip: time required to recover normal position
*Novel object test*
NO: total fish that crossed the novel object
*Anaesthesia test*
LSed: light sedation (fish exhibit a partial loss of reactivity)
TSed: total sedation (fish loss total equilibrium once turned by hand)
DSed: deep sedation (fish completely lost reflex to manipulation)
